# Factors associated with patients’ demand for low-value care: a scoping review

**DOI:** 10.1186/s12913-024-12093-7

**Published:** 2024-12-28

**Authors:** Gillroy R. L. Fraser, Mattijs S. Lambooij, Job van Exel, Raymond W. J. G. Ostelo, Frenk van Harreveld, G. Ardine de Wit

**Affiliations:** 1 Department of Health Economics and Health Services Research, National Institute for Public Health and the Environment (RIVM), P.O. Box 13720, Antonie van Leewenhoeklaan 9, Bilthoven, BA Netherlands; 2 Department of Health Sciences, Faculty of Science, Vrije Universiteit Amsterdam, Amsterdam Public Health Research Institute, Amsterdam, Netherlands; 3 Center for Prevention, Lifestyle and Health, Department Behaviour & Health, National Institute for Public Health and the Environment (RIVM), Bilthoven, Netherlands; 4 Erasmus School of Health Policy & Management, Erasmus University Rotterdam, Rotterdam, Netherlands; 5 Erasmus Centre for Health Economics Rotterdam (EsCHER), Rotterdam, Netherlands; 6 Department of Health Sciences, Faculty of Science, Vrije Universiteit Amsterdam, Amsterdam Movement Sciences Research Institute, Amsterdam, Netherlands; 7 Department of Epidemiology and Data Science, Department of Epidemiology and Data Science, Amsterdam University Medical Centre, Amsterdam University Medical Centre, Vrije Universiteit, Amsterdam, Netherlands; 8 Faculty of Social and Behavioral Sciences, University of Amsterdam, Amsterdam, Netherlands

**Keywords:** Low-value care, Cost-ineffective care, Overuse, Overdiagnosis, Patient preferences, Demand factors, Patient perspective

## Abstract

**Background:**

Low-value care is unnecessary care that contributes to inefficient use of health resources and constitutes a considerable proportion of healthcare expenditures worldwide. Factors contributing to patients’ demand for low-value care have often been overlooked and are dispersed in the literature. Therefore, the current study aimed to systematically summarize factors associated with patients’ demand for low-value care.

**Methods:**

In this scoping review, scientific articles were identified based on a search query conducted in Embase and Scopus. We identified articles using search terms related to low-value care and demand-related factors, published in peer-reviewed journals, and written in English or Dutch. The titles, abstracts, results, and conclusions were inspected to only include articles that were deemed relevant for this topic. From these articles we extracted text fragments that contained factors associated with patients’ demand for low-value care. Hereafter, a thematic analysis was applied to openly, axially, and selectively code textual fragments to identify themes within the data.

**Results:**

Forty-seven articles were included in this review. We identified eight core themes associated with patients’ demand for low-value care: cognitive biases, emotions, preferences and expectations, knowledge-related factors, socio-cultural factors, biomedical and care-related factors, economic factors, and factors related to the interaction with the healthcare provider. Within these core themes, thirty-three subthemes were identified. For example, risk aversion and anticipated regret aversion are sub-themes of cognitive biases, while consumerism and present and future income effects are sub-themes of economic factors.

**Conclusions:**

Through this review we provide a systematic overview of factors associated with the demand for low-value care. We found that patients’ demand for low-value care could relate to a multitude of factors that were clustered into eight core themes and thirty-three subthemes. To understand the demand for low-value care from the patient’s perspective in greater detail, future research should focus on the interaction between and importance of these factors in different care contexts.

**Supplementary Information:**

The online version contains supplementary material available at 10.1186/s12913-024-12093-7.

## Introduction

Some medical interventions are unnecessary and contribute to the inefficient use of health resources, by creating unnecessary costs and foregone health benefits [1, 2]. These interventions are often referred to as cost-ineffective care or low-value care [1, 2]. Examples of low-value care are routine imaging for nonspecific low-back pain, prescription of unnecessary antibiotics, and PSA-testing for patients older than 69 years [3–5]. Low-value care takes up a considerable amount of the healthcare expenditure in OECD countries. For example, at least 20% of the total healthcare expenditure in the US was spent on low-value care [1]. In the Netherlands, a fifth of the budget for acute care was spent on low-value care [1]. Furthermore, it is estimated that an average of 6% of the yearly healthcare expenditure for hospital care in the Netherlands is spent on cost-ineffective specialist medical care [6]. These estimates underline the importance of providing attention to low-value care to reduce unnecessary healthcare expenditure while simultaneously improving the quality of care. Therefore, researchers focused on various aspects of the low-value care phenomenon, including interventions developed to reduce low-value care [1, 5, 7–25] and on identifying factors contributing to the emergence of low-value care [24, 26–30].

Thus far, researchers concluded that low-value care is mainly driven by the interaction of system factors, clinician factors on the supply-side and patient factors on the demand-side [1, 5, 12, 26–28]. Most of the work focused on the supply-side or system factors and to a lesser extent on the demand-side factors of low-value care [5, 31, 32]. Studies on the supply-side factors reported that physicians rationalized the provision of low-value care in terms of upholding a good relationship with their patients, to satisfy patients’ demands, fear of missing a diagnosis and malpractice litigation, and perverse financial incentives of physicians in countries with fee for service payment models [5, 12, 26, 33–35]. Furthermore, studies on supply-side factors identified that use of low-value care was often the result of time pressure of physicians and a lack of time for shared decision making or educating patients about the low-value character of certain health services [5, 36–38].

While supply-side factors are often described in the literature, the existing scoping and systematic reviews on demand-side factors seem to have identified only a limited set of drivers to explain patients’ demand for low-value care [9, 10, 18, 19, 24, 39, 40]. One reason is the scope of these studies. For instance, a majority of these studies primarily focused on reducing low-value care in general, rather than identifying demand-related drivers specifically [9, 18, 19, 40, 41]. In addition, the studies aiming to identify drivers of low-value care, explored demand-related drivers alongside system and supply-related drivers, resulting in more general overviews of factors contributing to low-value care [26–28]. Finally, there are several studies that focused on one or a few demand drivers specifically, which have not all been covered in these existing reviews. For example, Perlman and Raeburn (2021) examine the association between various types of pain and risk-seeking preferences of patients in the context of financial and health-related decision-making. Other examples include consumeristic behaviour of patients to shop around for care, patients facing income related consequences due to sickness, patients’ trust in the provider, biased media reporting, emotions such as fear and insecurity, chronic symptoms, and pain-related factors have also been associated with low-value care demand, but have not been mentioned in the existing reviews [26, 27, 42–53].

Thus, because existing reviews had a different or more general scope and several studies on specific demand-side drivers have not been included in these reviews, the available evidence regarding factors explaining patients’ demand for low-value care is dispersed across the literature, and a systematic overview of demand-side factors is lacking. Therefore, the aim of this paper is to systematically summarize factors associated with patients’ demand for low-value care, as mentioned in the scientific literature.

## Materials and methods

We conducted a scoping review, which is a synthesis of systematically identified and mapped evidence on a certain topic [54, 55], in this case to study the existing knowledge about low-value care in the literature. We thus focus on systematically summarizing factors that are associated with patients’ demand for low-value care in the literature by following the PRISMA reporting guideline for scoping reviews [55].

### Operationalization of factors associated with low-value care demand

We defined factors associated with the demand for low-value care as determinants that drive patients to request and seek medical interventions that provide little to no health benefits and are potentially even harmful [1, 2, 7]. Within this review the term ‘’factors associated with patients’ demand’’ will be used interchangeably with other terms, such as ‘demand factors’, ‘patient drivers’ and ‘demand drivers’.

### Identification

**Fig. 1:**
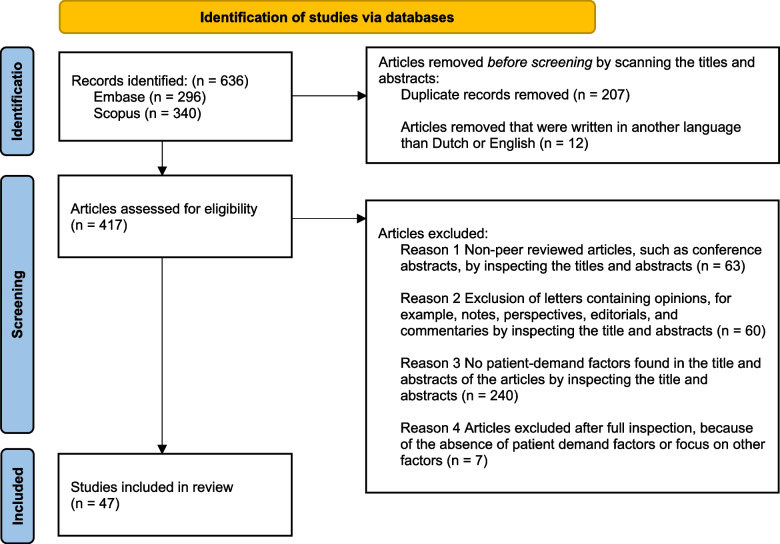
Prisma diagram for the selection of relevant articles

Data was collected by following the three stages of the Prisma guidelines [55, 56], which are the identification, screening and the inclusion stage (see Fig. 1). In the identification stage, a search strategy was constructed to identify relevant scientific articles for this study (see Appendix 1). The conducted search strategy consisted of two parts. In the first part of the identification stage, the focus was to identify articles relating to low-value care. Therefore, synonyms and terminology associated with low-value care were applied, such as: low-value care, low-value service, overdiagnosis, overuse, underuse, cost-ineffective care and unnecessary care (see Appendix 1) [1, 9, 26, 28, 57, 58].

In the second part of the identification stage, we added various factors related to patients’ demand as an extra requirement to our search strategy (see Appendix 1). Through this requirement we could identify articles within the topic of low-value care that also contain potential factors associated with patient’s demand for low-value care. Demand factors included in our search strategy were derived from a convenience sample of articles relevant to our topic. This sample consisted of 33 articles, of which 13 were selected that provided partial insights into low-value care demand drivers [1, 8, 9, 18, 24, 26–28, 30, 39, 57, 59, 60]. All findings from these papers were turned into search terms of factors that relate to demand for low-value care (see Appendix 1).

By combining these two groups of search terms, we performed the literature search on the 27th of May 2024. We decided not to have any time restrictions in this search, to cover all indexed papers regarding this topic. We selected the Embase.com and Scopus database for our literature search. Embase.com focuses primarily on journals containing medical subjects, including almost all journals covered by MEDLINE and PubMed. Therefore, a search concerning low-value care should provide accurate results in a medical oriented database. Additionally, we used Scopus because of its broad content (more than 40.000 journals) and inclusion of more than 330 disciplines, that was regarded to cover the non-medical factors (e.g. psychological, cultural and economic factors) associated with patients’ demand for low-value care.

### Screening and inclusion 

During the second stage of data collection, also referred to as the screening stage, we applied the following inclusion and exclusion criteria to assess the relevance of the identified articles for our study. We started by applying the exclusion criteria on the title and abstract of each article. One of our initial exclusion criteria was to remove articles written in a language other than Dutch or English. Subsequently, to uphold a base level of quality in the acquired data, non-peer reviewed articles such as conference abstracts were removed. Additionally, we excluded articles containing opinions, such as: letters, notes, perspectives, editorials, and commentaries. We read the title and abstract from the remaining articles and excluded articles that did not address factors associated with patients’ demand for low-value care. Following this, the remaining articles were fully inspected to verify if they contained factors associated with patients’ demand for low-value care. In cases of doubt, three authors (GRLF, GAdW and MSL) discussed whether certain factors in articles would be applicable for patients to demand low-value care, before excluding or including them.

### Data analysis: peer debriefing, checks and consensus making

A content analysis was conducted on the included articles. In the first stage of this analysis, three authors (GRLF, GAdW and MSL) identified and extracted textual fragments that contained factors associated with patients’ demand for low-value care. In the second stage, a thematic analysis was conducted. In this thematic analysis, extracted text fragments were labelled and categorized by using open, axial and selective coding [61]. During the coding process, primarily performed by the first author, regular peer-debriefing sessions were held with three co-authors (GAdW, JvE, MSL) to reflect on the manner in which coding was conducted[61]. In addition to these peer-debriefing sessions, three co-authors (GAdW, JvE and MSL) checked the open, axial, and selective codes. Differences in perspectives on, for example, how certain text fragments should be interpreted and coded, were discussed until consensus was reached. Afterwards, we (GRLF, GAdW, JvE and MSL) analysed the presence of the axial (subthemes) and selective codes (core themes) per article.

#### Data analysis: open coding

Open coding was the first step used to analyse the data. During this process all textual fragments containing factors associated with the demand for low-value care were inspected and summarized in single or multiple words and short sentences (open codes) [61]. Through open coding we attempted to capture and summarize the context of the text fragments without losing its true essence and meaning [61]. For example, the open code ‘family members pressure patients to demand unnecessary treatments’ was derived from the text fragment: ‘’Last, family members were referred to as sources of pressure by some participants, pushing patients to demand unnecessary treatments, or demanding on the patient’s behalf’’ [42]. In that example, pressure from family members was a factor that led patients to request low-value care. In certain cases, we assigned multiple open codes to text fragments that contained numerous factors.

#### Data analysis: axial coding

After converting various text fragments into open codes, the following step was to axially code the data. In this stage, four authors (GRLF, GAdW, JVE and MSL) created overarching subthemes for the open codes by grouping certain open codes together under a broader overarching subtheme [61]. By examining the meaning and context of each open code we identified similarities and constructed various subthemes or axial codes. We aimed to name these subthemes in accordance with similar concepts used in the literature when applicable. For instance, the subtheme ‘social network’ consists of open codes that drive patients to demand low-value care based on their social interactions and personal relationships with friends, family, and acquaintances. Therefore, the following open codes were grouped under this broader subtheme: ‘familial issues drive overuse’ and ‘family members pressure patients to demand unnecessary treatments’. The same approach was applied for the construction of the other subthemes.

#### Data analysis: selective coding

In the last coding stage, the number of subthemes was reduced by implementing selective codes. These are overarching core themes or dimensions that contain the corresponding subthemes and are considered the highest level of abstraction that subthemes can achieve [61]. For instance, subthemes relating to a certain emotional state such as: ‘fear and anxiety’, ‘perceived insecurity’, and ‘need for control’ were grouped under the core theme ‘emotions’.

## Results

During the identification stage, 636 articles were identified (i.e. 340 from Scopus and 296 from Embase). Of these articles, we excluded 207 duplicate articles, and 12 articles not written in Dutch or English, leaving 417 articles for screening. In the screening stage, 63 conference abstracts and articles that were not peer-reviewed were excluded. Next, after reading the abstracts, 60 articles containing opinions, and 240 articles that did not contain factors associated with the demand for low-value care were also excluded. The remaining 54 articles were subjected to a full inspection. After this full inspection, seven articles that did not contain and/or focus on factors associated with the demand for low-value care were excluded. Thus, a total of 522 articles were excluded based on our exclusion criteria for this study (see Fig. 1) and 47 articles were included in the review. Many of these articles are based on studies in high-income countries such as the United States, Canada, Australia, the Netherlands, Germany, France, Israel and China. However, studies from low- to middle-income countries, for example India, were also included. In addition, 15 of these studies focused on cancer-related diseases (See Table 1). Characteristics of the included articles are summarized in Table 1.

**Table 1:** Characteristics of the included studies

Study	Title	Country	Data collection	Participants	Condition examined	Low-value diagnostics ^a^	Low-value medical treatments ^a^
(Alber et al., 2017) [62]	Medical overuse and quaternary prevention in primary care – A qualitative study with general practitioners	Germany	In-depth Interviews	(*n* = 13) General Practitioners	No specific condition examined	-Diagnosis of abnormalities not related to disease -Unnecessary medical evaluation -Overtesting (not specified) -Overtreatment (not specified)	Not specified
(Bishop et al., 2017) [63]	Academic physicians' views on low-value services and the choosing Wisely campaign: A qualitative study	United States	Focus groups	(*n* = 7) Physicians from general internal medicine (*n* = 7) Physicians from emergency medicine (*n* = 7) Physicians from cardiology (*n* = 10) Physicians from hospital medicine	No specific condition examined	-X-rays for fractures -Head CTs for minor Head trauma -Chest CTs for dyspnea -Cultures for abscesses -Unnecessary lab work -Annual physicals -Pe-operative testing -Finger stick glucose testing -MRIs (by orthopedic surgeons) -Telemetry and CT scans for pulmonary embolism -Cardiac catheterization with stenting -Cardiac stress testing -Cardiac CT and MRI -Echocardiograms -Frequent lipid tests	-Antibiotics and anti-influenza medication for upper respiratory infections
(Born et al., 2019) [22]	Reducing overuse in healthcare: advancing Choosing Wisely	Not specified	Not specified	Not specified	No specific condition examined	-Unnecessary tests (not further specified)	-Urinary catheters -Antibiotic prescription
(Buist et al., 2016) [64]	Primary Care Clinicians’ Perspectives on Reducing Low-Value Care in an Integrated Delivery System	United States	Electronic cross-sectional survey	(*n* = 189) Clinicians	No specific condition examined	-High end imaging (computed tomography and magnetic resonance imaging) -Laboratory testing -Specialty referrals -PSA cancer screening -Over-screening for cervical cancer	-Antibiotics prescription for viral infections
(Carpenter et al., 2015) [65]	Overtesting and the Downstream consequences of Overtreatment: Implications of “Preventing Overdiagnosis” for Emergency Medicine	United states	Not specified	Not specified	No specific condition examined	-CT to diagnose pulmonary embolism -imaging for minor traumatic head injury -Cardiac stress testing	Not specified
(Duffin et al., 2020) [50]	Nonodontogenic Odontalgia Referred from the Temporal Tendon: A Case Report	United States	Case study	(*n* = 1) Patient	Temporal tendonitis	-Local anesthetic blockade -Diagnostic ultrasound -MRI	-Endodontic or extraction procedures -Inappropriate medication to manage pain or headaches -Unnecessary surgery temporomandibular joint dysfunction
(Edmeads et al., 1997) [66]	Strategies for diagnosing and managing medication-induced headache	Canada	Not specified	Not specified	Medication induced headache	-Laboratory investigations -Radiological investigations	-Medication induced headache caused by overuse of medicine (prescribed and Over the counter)
(Ellen et al., 2021) [42]	Understanding Physicians’ Perceptions of Overuse of Health Services in Oncology	Israel	Semi-structured interviews	(*n* = 15) Medical oncologists (*n* = 11) Radiation oncologists (*n* = 11) Hematologists (*n* = 4) Pediatric oncologists	Cancer	-Diagnostic tests such as imaging tests, tumor markers, genomic tests and mammograms, blood surveillance, and follow-up testing	-Treatment as an area of overuse (not specified) -Palliative care as an area where overuse tends to occur (not specified)
(Fifer et al., 2022) [67]	Improving adherence to guidelines for spine pain care: what tools could support primary care clinicians in conforming to guidelines?	United states	Semi-structured interviews	(*n* = 40) Primary care clinicians	Spine pain	-Imaging (MRI)	-Unnecessary pain medication (not specified) -Referrals for surgery (not specified)
(Gangathimmaiah et al., 2023) [68]	What works for and what hinders deimplementation of low-value care in emergency medicine practice? A scoping review	Not specified	Scoping review	(*n* = 167) studies	Emergency medicine practice	-Lumbar spine radiograph for atraumatic back pain -Chest radiograph for bronchiolitis -CT imaging for minor Head injury -CTPA for normal D-dimer	-Antibiotic use in sinusitis
(Gaver, 2022) [69]	Too Much Healthcare: The Harmful Combination of Overdiagnosis and Medical Overuse, Told and Untold Stories	Israel	Review	Not specified	No specific condition examined	-Thyroid, lung cancer screening in (low-risk patients), breast, and prostate cancer screening -CT-scans of the chests -Cardiac MRI scans -Spine MRIs -Brain MRIs	-Computed tomography colonoscopy
(Hersch et al., 2013) [51]	Women’s views on overdiagnosis in breast cancer screening: a qualitative study	Australia	Focus groups	(*n* = 50) Women aged 40–79 years	Breast cancer	-Mammography screening	Not specified
(Hofmann, 2020) [70]	Biases distorting priority setting	Not specified	Literature content analysis	Not specified	No specific condition examined	-Cancer screenings	-Overdiagnosis in cancer screening programs
(Hofmann, 2021) [71]	Internal barriers to efficiency: why disinvestments are so difficult. Identifying and addressing internal barriers to disinvestment of health technologies	Not specified	Not specified	Not specified	No specific condition examined	Not specified	Not specified
(Hudgins and Rising, 2016) [72]	Fear, vulnerability and sacrifice: Drivers of emergency department use and implications for policy	United States	Case study	(*n* = 1) Patient	Frequent headaches	Not specified	Not specified
(Jensen et al., 2020) [53]	From Overdiagnosis to Overtreatment of Low-Risk Thyroid Cancer: A Thematic Analysis of Attitudes and Beliefs of Endocrinologists, Surgeons, and Patients	United States	Semi-structured interviews	(*n* = 12) Surgeons (*n* = 12) Endocrinologists (*n* = 10) Patients with papillary thyroid cancer	Low-risk Thyroid Cancer	-Overdiagnosis of patients with thyroid cancer (not specified)	-Unnecessary thyroidectomy -Unnecessary Thyroid nodule biopsy
(Kangovi et al., 2013) [52]	Understanding Why Patients Of Low Socioeconomic Status Prefer Hospitals Over Ambulatory Care	United States	Interviews	(*n* = 40) Urban low-SES patients	No specific condition examined	Not specified	Not specified
(Kazemi et al., 2024) [73]	Understanding Treatment Decision-Making in Older Women With Breast Cancer: A Survey-Based Study	United States	Explanatory cross-sectional survey	(*n* = 29) Women ≥ 70 years of age	Breast cancer	Not specified	-Axillary staging with sentinel lymph node biopsy (SLNB) -Adjuvant radiotherapy following lumpectomy
(Kherad et al., 2020) [40]	The challenge of implementing Less is More medicine: A European perspective	Not specified	Review	Not specified	No specific condition examined	-Routine cancer screening (further not specified)	-Antibiotics given to children -Antibiotics for acute respiratory infections
(Kool et al., 2020) [5]	Identifying and de-implementing low-value care in primary care: the GP’s perspective—a cross-sectional survey	The Netherlands	Cross-sectional survey	(*n* = 182) General practitioners	No specific condition examined	-Lumbosacral spine X-rays for low-back pain -low back pain imaging -X-rays for osteoarthritis -Echocardiography for Chest pain -Prostate specific antigen test -Vitamin B12 Laboratory tests without an evidence-based indication	-Antibiotics prescription -Benzodiazepine’s prescription -Opioid’s prescription -Vitamin-supplements prescription -minor cosmetic surgery
(McCaffery et al., 2016) [74]	Walking the tightrope: communicating overdiagnosis in modern healthcare	Not specified	Not specified	Not specified	No specific condition examined	-Overdiagnosis / screening of cancer -Prostate specific antigen testing -Overdiagnosis breast screening	-Elective prostate surgery (management -Elective surgery for non-invasive breast cancer
(Mott et al., 2021) [46]	Medical Maximizing–Minimizing Preferences in Relation to Low-Value Services for Older Women with Hormone Receptor-Positive Breast Cancer: A Qualitative Study	United States	Semi-structured interviews	(*n* = 30) Women ≥ 70 years of age	Breast cancer	-Breast cancer testing	Breast cancer treatment such as surgery: -Mastectomy, -Lumpectomy -Sentinel lymph node biopsy -Chemotherapy -Radiotherapy
(Munoz-Plaza et al., 2016) [75]	Integrating qualitative research methods into care improvement efforts within a learning health system: addressing antibiotic overuse	United States	In-depth interviews	(*n* = 6) Primary care physicians (*n* = 3) Urgent care physicians	Acute sinusitis	-Computed tomography imaging	-Inappropriate antibiotic prescription for sinusitis
Pathirana et al., 2017) [26]	Mapping the drivers of overdiagnosis to potential solutions	Australia	Literature review	(*n* = 41) Studies	No specific condition examined	Not specified	Not specified
(Paz-Martin and Arnal-Velasco, 2023) [76]	Can we nudge to reduce the perioperative low-value care? Decision making factors influencing safe practice implementation	Not specified	Review	Not specified	No specific condition examined	-Screening tests (not further specified)	-Inappropriate consumption of Antibiotics -Hospital overutilization versus primary care -Cholecystectomy in asymptomatic cholelithiasis
(Perlman and Raeburn, 2021) [49]	The association between willingness to take risks with type-specific intensity of chronic pain	United States	Online Survey	(*n* = 82 Participants (public) chronic pain sample (*n* = 92) Participants (public) representative sample	No specific condition examined	Not specified	Not specified
(Pickles et al., 2015) [77]	Doctors’ approaches to PSA testing and overdiagnosis in primary healthcare: a qualitative study	Australia	Semi-structured interviews	(*n* = 32) General practitioners	Prostate cancer	-Prostate specific antigen screening	Not specified
(Pickles et al., 2021) [78]	Preferences for More or Less Health Care and Association With Health Literacy of Men Eligible for Prostate-Specific Antigen Screening in Australia	Australia	Survey	(*n* = 4793) Men	Prostate cancer	-Prostate-specific antigen screening	Not specified
(Podder et al., 2019) [79]	Middle-aged man who could not afford an angioplasty	India	Case study	(*n* = 1) Patient	Coronary artery disease	Not specified	-Percutaneous coronary intervention (PCI)
(Rapoport, 2008) [43]	Medication Overuse Headache—Awareness, Detection and Treatment	Not specified	Review	Not specified	Medication overuse headache	Not specified	-Medication overuse headache caused by frequent use of (prescribed) acute medications that are ineffective in aborting the headache process
(Ropers et al., 2023) [80]	Diagnostic testing in children: A qualitative study of pediatricians' considerations	The Netherlands	In-depth and semi-structured interviews	(*n* = 20) Dutch pediatricians	No specific condition examined	Not specified	Not specified
(Rozbroj et al., 2021) [39]	How do people understand overtesting and overdiagnosis? Systematic review and meta-synthesis of qualitative research	Not specified	Systematic review and meta-synthesis of qualitative research	(*n* = 21) Studies	Cancer such as: -Thyroid -Cervical -Breast -Prostate -Lung Other conditions: -Osteoporosis -Attention deficit hyperactivity disorder -Neonatal issues	-Cancer screening -Osteoporosis screening -Attention deficit hyperactivity disorder -Neonatal population screening	Not specified
(Rudin et al., 2022) [81]	Addressing the Drivers of Medical Test Overuse and Cascades: User-Centered Design to Improve Patient–Doctor Communication	United States	Focus groups and 1:1 design meetings	(*n* = 15) General practitioners (*n* = 22) Patients	No specific condition examined	-Imaging tests such as: MRI’s and X-rays for low back pain -Laboratory tests (not specified)	Not specified
(Salm et al., 2023) [82]	Analysis of the key themes in the healthcare of older people with multimorbidity in Germany: a framework analysis as part of the LoChro trial	Germany	Framework analysis	(*n* = 6) Multimorbid older patients	No specific condition examined	Not specified	Not specified
(Scherer et al., 2020) [47]	Medical Maximizing-Minimizing Predicts Patient Preferences for High- and Low-Benefit Care	United States	Online survey	(*n* = 785) Public	No specific condition examined	-MRI for nonacute, low back pain -Full-body CT scan for asymptomatic, healthy person -CT scan for minor head injury without red-flag symptoms -CT scan for mild head injury to 5-year-old child with headache as only symptom -Take 5-year-old child to emergency department immediately for fever -Take 3-year-old child to emergency department for vomiting (fluid intake, normal urination)	-Surgery for plantar fasciitis -Opioid for chronic knee pain that is not controlled by Ibuprofen -Continued colonoscopy after age 80 years -Antacid medication for colicky baby who is otherwise healthy and gaining weight
(Sicsic et al., 2018) [83]	Women’s Benefits and Harms Trade-Offs in Breast Cancer Screening: Results from a Discrete-Choice Experiment	France	Discrete-choice experiment	(*n* = 812) Women aged 40 to 74 years	Breast cancer	-(False-positive) Mammography	Not specified
(Siedlikowski et al., 2018) [84]	Scrutinizing screening: a critical interpretive review of primary care provider perspectives on mammography decisionmaking with average-risk women	Not specified	Critical interpretive review	(*n* = 9) Articles	Breast cancer	-Mammography for women with average-risks	Not specified
(Skolarus et al., 2021) [85]	Learning from the “tail end” of deimplementation: the case of chemical castration for localized prostate cancer	United States	Interviews	(*n* = 19) Urology providers (*n* = 12) Patients	Prostate cancer	-Prostate specific antigen screening -High-frequency ultrasound ablation -MRI (not specified)	-Prostate biopsy -Cryotherapy -Androgen deprivation therapy -Chemical castration
(Strobel et al., 2023) [86]	Factors influencing defensive medicine‐based decision‐making in primary care: A scoping review	Not specified	Scoping Review	(*n* = 13) Publications	No specific condition examined	Not specified	Not specified
(Sutkowi-Hemstreet et al., 2015) [87]	Adult Patients’ Perspectives on the Benefits and Harms of Overused Screening Tests: a Qualitative Study	United States	Semi-structured interviews	(*n* = 50) Patients	No specific condition examined	-Prostate cancer screening -Colon cancer screening -Osteoporosis screening -Cardiovascular disease screening	Not specified
(Taylor et al., 2018) [48]	Surgical Management of Lobular Carcinoma In Situ: Analysis of the National Cancer Database	United states	Data from the National Cancer Database	(*n* = 30,105) Patients	Lobular carcinoma in situ	Not specified	-Unilateral mastectomy -Bilateral mastectomy
(van Egmond et al., 2019) [88]	Factors influencing current low-value follow-up care after basal cell carcinoma and suggested strategies for de-adoption: a qualitative study	The Netherlands	Semi-structured interviews and focus groups	(*n* = 18) Dermatologists (*n* = 17) Patients	Basal cell carcinoma	-Follow up visits after skin cancer diagnosis	Not specified
(van Egmond et al., 2021) [89]	What do patients and dermatologists prefer regarding low-risk basal cell carcinoma follow-up care? A discrete choice experiment	The Netherlands	Survey including a discrete choice experiment	(*n* = 371) Basal cell carcinoma patients	Basal cell carcinoma	-Basal cell carcinoma follow-up visits	Not specified
(Vercellini et al., 2015) [90]	Reducing low-value care in endometriosis between limited evidence and unresolved issues: a proposal	Not specified	Systematic literature search	Not specified	Endometriosis	Not specified	-Laparoscopy for diagnostic purposes -Hormonal therapies used for curative purposes and not for pain relieve
(Wammes et al., 2014) [44]	Is the role as gatekeeper still feasible? A survey among Dutch general practitioners	The Netherlands	Survey	(*n* = 600) General practitioners	No specific condition examined	-CT-Scan for headaches -MRI for headaches -Consultation demanded after providing an explanation by the general practitioners’ assistant on the phone	Not specified
(Wang et al., 2021) [91]	Trends in Breast Cancer Treatment De-Implementation in Older Patients with Hormone Receptor-Positive Breast Cancer: A Mixed Methods Study	United States	Mixed methods	(*n *= 263) Women for quantitative study (*n* = 12) structured interviews study	Breast Cancer	Not specified	-Lumpectomy (sentinel lymph node biopsy) -Post-Lumpectomy radiotherapy
(Yin et al., 2019) [45]	Pattern of antibiotic prescribing and factors associated with it in eight village clinics in rural Shandong Province, China: a descriptive study	China	Semi- structured interview	(*n* = 16) Doctors and deputy director	Upper Respiratory Tract Infections such as: -Common cold -Acute sinusitis -Acute pharyngitis -Acute Tonsillitis -Acute Laryngitis -Tracheitis), -Acute obstructive laryngitis -Epiglottitis -Acute upper respiratory infections of multiple and unspecified sites	Not specified	-Antibiotic prescription

^a^see article for detailed information about low-value context

In total, 219 text fragments containing factors related to demand for low-value care were extracted from the 47 included articles. Through the process of open, axial, and selective coding we identified the following eight core themes in the literature: 1) cognitive biases; 2) emotions; 3) preferences and expectations; 4) knowledge-related factors; 5) socio-cultural factors; 6) economic factors; 7) biomedical and care-related factors; and 8) factors related to the interaction with the healthcare provider. These core themes were broken down into 33 subthemes that explain in greater detail why patients may demand low-value care. We constructed an overview of the identified themes and the presence of each core and subtheme per article in Appendix 3.

In the following section, the various identified core and subthemes are explained and discussed. Additionally, we indicated in Appendix 3 how often each theme occurs in the 47 included papers. Thereafter, we provide examples of open codes assigned to each subtheme. Appendix 2 contains the full process of coding, including the textual fragments and the open, axial, and selective codes assigned to them.

### Cognitive biases

We identified ‘cognitive biases’ (covered in 31 of the 47 included articles) as a core theme contributing to patients’ demand for low-value care. A cognitive bias is a systematically occurring tendency to think, act or feel in a certain manner, caused by processing and interpreting information derived from individual experiences and preferences [70, 92]. Within this core theme, ten distinct types of cognitive biases were identified as subthemes.

The first subtheme, ‘asymmetry of risks and benefits’ (covered in 13 of the 47 included articles), is a tendency of patients to overestimate the benefits and underestimate the risks of low-value care treatments (Hofmann, 2020). Three examples of open codes assigned to this theme are: ‘to understand risks and benefits in very unbalanced and biased ways’, ‘over-relying on care and not knowing the limits and harms’, and ‘overestimating the benefit that more treatment leads to improved survival’ [40, 70, 74].

The second subtheme, ‘extension bias’ (covered in 13 of the 47 included articles), is the tendency of patients to perceive more healthcare as better than less healthcare [70]. Patients incentivized by this bias can demand excessive, unnecessary, and even harmful care. Three examples of open codes that were clustered in this theme are: ‘the public’s view that more is better’, ‘a tendency to think that more care is better than less care’, and ‘pursuing more versus less medical testing and treatments’ [22, 46, 70].

The third subtheme, ‘imperative knowledge bias’ (covered in 11 of the 47 included articles), is a tendency of patients to gain more information related to their health status, because this is better than being ignorant. Therefore, patients tend to think that ‘’to know is better than not to know’’ [70]. This bias occurs primarily in the field of diagnostics, and usually leads to overdiagnosis. The following three examples are open codes assigned to this subtheme: ‘thinking that early detection is better than later detection', 'early diagnosis is part of a preventive strategy', and 'tests and screenings enable early interventions' [39, 40, 70].

The ‘imperative action bias’ (covered in 8 of the 47 included articles) was found as the fourth subtheme. Patients with this bias feel obliged to act, because ‘’action is better than inaction’’ [71]. Three examples of open codes assigned to this subtheme are: ‘something must happen, because action is better than inaction’, ‘good medicine means taking action’, and ‘patients give credit for trying to do something’ [40, 71].

‘Risk aversion’ (covered in 7 of the 47 included articles), is a tendency of patients to avoid or reduce uncertainties, dangerous situations, and risks as much as possible [48, 70, 91]. The open codes attributed to this subtheme are: ‘aversions to dangers or uncertainty’, ‘elevated risk for cancer development may result in proactive behaviour’, and ‘eradicating remaining cancer reduces the risk of disease spread’ [48, 70, 91].

‘Anticipated regret aversion’ (covered in 6 of the 47 included articles) includes instances where patients strive to avoid possible regret in the future by demanding unnecessary care in the present [70, 71]. We assigned the following three examples of open codes to this subtheme: ‘to undergo procedures just in case', better safe than sorry’, and ‘conducting an early diagnosis to avoid diagnosing a disease too late’ [26, 42, 70, 71].

The ‘confirmation bias’ (covered in 4 of the 47 included articles) occurs when patients seek or interpret medical information or care recommendations that correspond to their viewpoints, and disregard or dispute contradicting recommendations and information [78]. Furthermore, patients influenced by this bias can prefer more healthcare than needed, and ignore, reject, or give less attention to information about the harms of screening and overdiagnosis [78]. Three examples of open codes that were assigned to this subtheme are: ‘demanding care until told what they want to hear’, ‘to discount advice that is discordant with one’s own beliefs’, and ‘interpreting information to confirm existing positions and ignore or dispute contradictory information’ [42, 46, 78].

The eighth subtheme ‘loss aversion’ (covered in 4 of the 47 included articles), occurs when patients overvalue low-value care options and experience discomfort with losing a preferred care option [40, 71]. These patients tend to continue demanding low-value care even when physicians do not recommend this, because the losses loom larger than the gains. Three examples of open codes used to construct this theme are: ‘the loss of an option is greater than the utility associated with acquiring it', 'we feel uncomfortable with losing what we have, because we attribute unreasonable value to what we have’, and ‘to bristle at recommendations that limit choice’ [40, 70, 71].

‘Anchoring effect’ (covered in 2 of the 47 included articles) occurs when patients rely on initially found or received information, instead of relying on evidence that is of a higher quality but possibly more difficult to find. The open codes connected to the subtheme ‘anchoring effect’ are: ‘citing information which is not reputable', and 'relying on initial information and not on high quality evidence’ [70].

The ‘prominence effect’ (covered in 1 of the 47 included articles) was the last observed cognitive bias in the data. Patients can perceive medical decision-making as complex and challenging because of numerous factors that must be considered. Because of this, patients may try to resolve this complexity by focusing on a single aspect that for some reason is salient to them [70]. The open code attributed to this subtheme is: ‘the tendency to focus on one dominant factor for decision-making’ [70].

### Preferences and expectations

This core theme (covered in 27 of the 47 included articles) contains personal convictions and past experiences of patients associated with the demand low-value care. The two subthemes included in this theme are ‘beliefs’ and ‘experiences’.

Patients’ ‘beliefs’ (covered in 22 of the 47 included articles) are formed by personal convictions, which function as cornerstones that shape patients’ attitudes, preferences, and expectations towards medical care. These beliefs tend to make patients susceptible to perceive low-value care as a default or necessary treatment, which causes these patients to demand low-value care [46, 47, 53]. ‘The idea of overdiagnosis challenged beliefs about cancer’, ‘popular deep-seated beliefs in overdiagnosis and unnecessary testing’, and’beliefs appeared to influence the acceptance of overuse’ are three examples of open codes grouped under the subtheme ‘beliefs’ [26, 39, 51].

The ‘experiences’ (covered in 11 of the 47 included articles) of patients refer to past events that shape or influence their preferences and expectations to demand medical care [69, 87, 88]. When current or previous healthcare providers (repeatedly) provided certain care, an expectation may be formed [83, 87, 88]. This makes it harder for patients to accept that previously received care is not needed and to adapt their demand for low-value care. Providers may tend to avoid these discussions and, hence, continue to provide low-value care when patients demand care based on past experiences [88]. The three open codes assigned to this subtheme are: ‘experiences appeared to influence the acceptance of overuse’, ‘experiences influenced patients to ask for medical tests’, and ‘providing annual follow-up visits in the past creates an unnecessary habit for patients’ [39, 81, 88].

### Emotions

‘Emotions’ (covered in 24 of the 47 included articles) was a third core theme that we identified. Research in the domain of psychology has long established that many types of (health-related) behaviours are to a significant extent driven by emotions [93]. While the range of emotional influences on health-related behaviour is broad, in the context of low-value care three subthemes were found to drive demand: ‘perceived insecurity’, ‘fear and anxiety’, and ‘need for control’.

‘Perceived insecurity’ (covered in 18 of the 47 included articles) causes patients to feel uncertain about their health status [42, 51, 74]. Patients then may wish to reassure themselves by demanding care, including low-value care. Three examples of open codes assigned to this subtheme are: ‘screening is an opportunity to reassure that everything is all right’, ‘removing cancer provides peace of mind’, and’patients tend to over-rely on tests as a means of reassurance' [26, 51, 53].

Second, the subtheme ‘fear and anxiety’ (covered in 17 of the 47 included articles) refers to being afraid of illnesses, overlooking potential diseases, or possible outcomes of diseases. To reduce these fears and anxieties, patients are more likely to demand low-value care. Three examples of open codes assigned to this subtheme are: 'the fear of a potential disease dominates patients' thoughts', 'higher levels of anxiety result in surgical treatment options’, and ‘a yearly check-up would reduce their anxiety' [53, 71, 88].

Third, ‘need for control’ (covered in 3 of the 47 included articles) may also drive patients to pursue low-value care. Patients strive to assert control over their situation by displaying an active role in their decision-making process [42]. For example, patients might try to gather as much information as they can regarding their situation and act upon it by demanding care, even when this is unnecessary [42]. The three open codes connected to this subtheme are: ‘to assert control over their situation by gathering information and demanding unnecessary care', ‘patients prefer defensive medicine to increase perceived control’, and ‘knowing would give patients a sense of control’ [42, 76, 87].

### Knowledge-related factors

The core theme’knowledge-related factors’ (covered in 18 of the 47 included articles) was constructed out of all information and education-related factors that drive patients to demand low-value care. The following four subthemes were constructed: ‘Limited health literacy, ‘not accepting the concept of overuse’, ‘unawareness’, and ‘over-informed’.

‘Limited health literacy’ (covered in 9 of the 47 included articles) arises when patients lack sufficient ability to comprehend information about low-value care treatments and services. For example, patients lacking sufficient knowledge, tertiary education, or (medical) health literacy can demand low-value care [42, 78]. However, there are also examples of patients with higher education levels demanding low-value care [48]. Three examples of open codes linked to this subtheme are: ‘patients without sufficient knowledge take ideas out of context’, ‘lack of health literacy drives overuse’, and 'limited health literacy is associated with incorrect care perceptions' [39, 42, 79].

The subtheme ‘not accepting the concept of overuse' (covered in 8 of the 47 included articles) encompasses tendencies of patients to reject or question the credibility of evidence-based recommendations and overuse messaging to avoid low-value care interventions, such as overtreatment and overdiagnosis [39]. Three examples of open codes assigned to this subtheme are: ‘patients do not accept overuse’, ‘questioning and disagreeing with the concept of overdiagnosis’, and ‘patients rejected overuse messaging, questioned its legitimacy and viewed it as unsound’ [22, 39, 51].

In some cases, patients lack realization and information that certain treatments have no or only marginal health benefits and are possibly even harmful. Because of their ‘unawareness’ (covered in 7 of the 47 included articles) patients are often surprised to learn about the downsides of interventions characterized as low-value care. Three examples of open codes allocated to this subtheme are: ‘patients are unaware about the risks involved in medication use’, ‘limited awareness of overdiagnosis’, and 'the idea of overdiagnosis was surprising’ [51, 66].

The subtheme ‘over-informed’ (covered in 3 of the 47 included articles) refers to instances where patients possess too much information, resulting in a sense of data overload and wrongful healthcare decision-making. The following three examples are open codes assigned to this subtheme: ‘over-informed patients demand low-value care’, 'gathering all possible information can cause harm', and ‘patients have an intrinsic value to be as informed as possible’ [39, 42].

### Socio-cultural factors

The ‘socio-cultural’ core theme (covered in 18 of the 47 included articles) was established to contain three subthemes: ‘stage of life’, ‘entitlement to care’, and ‘social network’.

‘Stage of life’ (covered in 9 of the 47 included articles) concerns age- and life phase related factors that result in patients’ low-value care demand. For example, some individuals perceive that being young of age justifies more aggressive unnecessary treatment [46]. Three examples of open codes corresponding to ‘stage of life’ are: ‘taking a more aggressive approach if they were younger and wanted to fulfil life goals', 'ageing contributes to using too much medicine’, and 'being more inclined to pursue treatments if they were much younger' [26, 46, 91].

‘Social network’ (covered in 9 of the 47 included articles) encompasses instances where relatives and acquaintances pressure or encourage patients to seek low-value care [42, 68, 80]. Three examples of open codes assigned to this subtheme are: ‘cultural and familial issues drive overuse', 'family members pressure patients to demand unnecessary treatments’, and ‘parents could cause their children to undergo diagnostic testing’ [42, 68, 80].

‘Entitlement to care’ (covered in 4 of the 47 included articles) is the idea that patients perceive access to any requested healthcare as their right. For instance, patients justify demanding low-value care because they feel they pay a substantial amount for their healthcare insurance and, therefore, should be entitled to receive care when they demand it. Because of this, patients have higher expectations of care and are more willing to consume unnecessary and excessive healthcare [40]. In other cases, the existence of public standards or social norms determine this sense of care entitlement [26, 47]. Three examples of open codes assigned to this subtheme are: ‘having huge expectations of the expensive healthcare system’, 'perceived social norms determine medical preferences', and 'patients experience healthcare as a right' [40, 44, 47].

### Economic factors

The core theme ‘economic factors’ (covered in 16 of the 47 included articles) was constructed to contain factors relating to the consumption of low-value care for financial reasons or to satisfy economically oriented needs of patients. The following four subthemes were included in this core theme, ‘marketing’, ‘consumerism’, ‘present and future income effects’, and ‘insurance coverage’.

‘Consumerism’ (covered in 8 of the 47 included articles) refers to the idea that healthcare is perceived as a consumption good and patients can, therefore, shop around by visiting various healthcare providers to receive their desired care [42]. Three examples of open codes clustered under this subtheme are: ‘shopping around for physicians to receive preferred care’, ‘culture of healthcare consumption’, and ‘consumerism among patients drives unnecessary care’ [42, 44, 81].

‘Marketing’ (covered in 6 of the 47 included articles) occurs when information is presented in such a manner that it generates profits for the presenting actor and causes patients to demand low-value care [74]. In some cases, campaigns or advertisements expose consumers solely to the potential benefits of low-value care services [74]. In other cases, patients are misled by awareness campaigns about certain medical conditions overemphasizing the number of patients suffering from a certain disease. Consequently, in the eyes of the public, some diseases are perceived as more common or more serious than they actually are [74]. The following three examples of open codes illustrate this issue: ‘selling the idea that seeking medical care is the key to maintain wellness', 'pharmaceutical and device manufacturers maximize product sales by funding incorrect disease awareness campaigns’, and ‘the use of and promotion of sensitive tests that often lead to detection of minor abnormalities’ [26, 40, 74].

‘Present and future income effects’ (covered in 5 of the 47 included articles) concerns the need of patients to function at work to earn money and support their family. For this reason, patients are more likely to request low-value care when they are injured or sick [43, 45]. Three examples of open codes assigned to ‘present and future income effects’ are: ‘the need to function at work or home’, ‘curing the primary breadwinners of a family as soon as possible’, and 'If it's found earlier, It's going to be easier to handle financially’ [43, 45, 87].

‘Insurance coverage’ (covered in 2 of the 47 included articles) concerns the behaviour of demanding low-value care by patients because it is covered by insurance and, therefore, they do not directly bear the financial consequences of care use. The two open codes that were used to construct 'insurance coverage' are: 'having insurance drove him to get care', and 'insurance coverage can influence patients' decisions' [72, 87].

### Biomedical and care-related factors

The core theme ‘biomedical and care-related factors’ (covered in 18 of the 47 included articles) was constructed to include subthemes on the intersection of biology, medicine and medical care. For this reason, the following subthemes were clustered into this core theme: ‘severity and number of health threats’, ‘maximization of length and/or quality of life’, ‘duration of symptom or illness’, ‘pain’, and ‘ease of use’.

‘Severity and number of health threats’ (covered in 11 of the 47 included articles) refers to the fact that patients experiencing multimorbidity, severe symptoms or illnesses have an increased tendency to demand low-value care [47, 67, 72, 76]. Three examples of open codes assigned to this subtheme are: ‘the severity of a migraine attack can cause unnecessary care', ‘patient comorbidity may contribute to low-value care use’, and 'perceived severity of the health threat determine medical preferences' [43, 47, 76].

‘Maximization of length and/or quality of life’ (covered in 7 of the 47 included articles) concerns the wish of patients to live as long as possible at any cost, and to maintain or improve their current quality of life [42, 70, 87]. Some patients are even willing to improve their survival chances by consuming unnecessary and excessive healthcare even if this means reducing their quality of life and experiencing potential harms of care [39, 73]. This subtheme was was often found in cancer-related articles and mentioned as a motivation by patients to use all means and interventions possible [42, 46, 73, 83, 87]. Three examples of open codes assigned to this subtheme are: ‘the idea to extend life at all costs regardless of the quality of life’, 'a strong urge to save lives no matter what’, and ‘patients accepted that overuse can cause suffering, loss of quality of life, and loss of income' [39, 42, 70].

The subtheme ‘duration of illness or symptom’ (covered in 2 of the 47 included articles) encompasses the idea that a persisting or reoccurring medical problem, symptom, or disease, contributes to excessive demand for low-value care [49, 82]. For example, patients experiencing a symptom for an extended period of time have a higher likelihood to demand harmful or unnecessary care [49]. The two open codes assigned to this subtheme are: ‘Chronic symptoms contribute to patients' harmful and risky decision-making’, and ‘patient needs in terms of continuing pathologies led to frequent doctor visits’ [49, 82].

‘Pain’ (covered in 2 of the 47 included articles) experienced by patients was observed to be a factor for the prescription of inappropriate medication and unnecessary treatment by the provider [50]. The two open codes attributed to this subtheme are: ‘conducting unnecessary procedures because of pain experienced by the patient’, and ‘the pain drove him to get care’ [50, 72].

‘Ease of use’ (covered in 2 of the 47 included articles) concerns the idea of demanding care because it is convenient (e.g., nearby), time efficient, or perceiving other options as uncomfortable by the patient [67]. These reasons may contribute to the appeal and demand of various types of low-value care by patients [67, 87]. This subtheme was often related to interventions that were perceived as quick and non-invasive by patients, such as excessive screening and over-testing. The two open codes related to ‘ease of use’ are: ‘lacking time for low-intensity treatments like physiotherapy’, and ‘the willingness to do convenient, time-efficient, and comfortable testing’ [67, 87].

### Interaction with the healthcare provider

The core theme ‘interaction with the healthcare provider’ (covered in 11 of the 47 included articles) includes subthemes related to the interaction with health care providers that result in the demand for low-value care. The subthemes included into this theme are: ‘lack of trust in the provider’, and ‘acceptance of care recommended by the provider’.

‘Lack of trust in the provider’ (covered in 8 of the 47 included articles) was found as a subtheme for patients to demand low-value care. It is hypothesized that patients sometimes have less trust in the diagnostic capabilities of the primary care physician than of medical specialists and, therefore, ask to be referred to a medical specialist. Three examples of open codes connected to this subtheme are: ‘patients lack confidence in their physicians' abilities’, ‘having more confidence in a dermatologist than in a GP’, and ‘feeling more at ease when referred instead of examined by the GP’ [44, 66, 88].

‘Acceptance of care recommended by the provider’ (covered in 5 of the 47 included articles) includes instances where patients adhered to low-value care recommendations provided by their healthcare providers [69, 81, 87–89]. Three examples of open codes clustered under this subtheme are: 'getting any test the doctor recommends', ‘accepting unnecessary follow-up visits recommended by the previous dermatologist’, and ‘physicians’ recommendations greatly influenced patients’ decisions’ [81, 87, 88].

## Discussion

This scoping review systematically summarized factors associated with demand for low-value care. The identified factors were categorized into eight core themes and 33 subthemes. These core themes and subthemes provide a deeper understanding of why patients demand low-value care. Core themes originating from psychology were most common in the included literature. For instance, the three core themes ‘cognitive biases’, ‘emotions’, and ‘preferences and expectations’ together cover approximately half of the identified subthemes (i.e., 15 of 33 subthemes).

In addition to the factors that we have described in our review, several studies highlighted socio-demographic characteristics of patients potentially associated with demanding low-value care, including sex, education, race, socioeconomic status and certain occupations of patients [48, 76, 78, 83, 87, 89, 94]. This evidence, however, is mixed and needs further study. To name an example, gender may appear as a relevant factor in context specific studies where patients suffering from either breast cancer, endometriosis, or prostate cancer demand low-value care. However, in such context-specific studies, gender should be seen as a descriptor of the patient group or a confounding variable rather than a meaningful predictor of demand. It is no surprise that low-value care among patients suffering from breast cancer and endometriosis is most often demanded by female patients [83], while low-value care among patients suffering from prostate cancer is uniquely demanded by male patients [94]. In other cases, there is conflicting evidence between studies. For instance, being (un)employed and having (lower or) higher level of education were associated with lower and higher odds of demanding for low-value care in different studies [48, 78, 83, 94]. These varying results may, for instance, stem from differences in setting and (medical) conditions examined in these studies, and one should be cautious when interpreting the results of these studies. Therefore, we decided not to include ‘socio-demographic’ factors in our review.

The present study has some limitations. First, the current list of factors, although extensive may not be exhaustive. This may originate from our search strategy or lack of evidence on specific determinants in current literature, specifically in relation to patient characteristics. While constructing our search query we used keywords and factors found in articles from a convenience sample of papers that included low-value care demand drivers. Therefore, alongside broad patient factors, we also included several specific search terms, as for example fear, perceived insecurity, cognitive bias, and health beliefs. Including these specific search terms may have affected the comprehensiveness or composition of the results, and could perhaps explain the dominant presence of the core themes ‘cognitive bias’, ‘emotions’ and ‘preferences and expectations’ in the included literature.

Secondly, while the study provides insights into several factors that may relate to the structure of healthcare systems in different countries, we believe there still is room for further exploration of this topic. Factors like consumerism, entitlement to care, and insurance coverage were more dominant in studies conducted in Germany and the Netherlands, which have broad coverage of healthcare for all citizens [44, 62, 80, 82]. This indicates that systems providing universal coverage and access may see higher low-value care demand. On the other hand, some of the studies conducted in the United States focused more on concerns for out-of-pocket-costs, which relates to the subtheme present and future income effects [72, 87]. This may indicate that demand for low-value care is also present in systems that rely on private insurance and out-of-pocket payment for care. In our analysis of the included literature, we did not observe clear distinct patterns in drivers for low-value care demand across countries and healthcare systems. This may be due to the limited number of articles included from countries like Canada, Israel, Australia, France, China and India. Another reason may be the aims of these studies. In many cases, studies focused more on understanding perceptions of patient behaviour in specific clinical or disease-related settings, and less on how these perceptions are associated with the local healthcare system.

A third limitation is that 15 of the 47 included studies concerned cancer care. We performed a sensitivity analysis to grasp the consequences of this strong representation for our results. Excluding these 15 articles would primarily diminish but not entirely remove the following subthemes: ‘risk aversion’, ‘confirmation bias’, ‘beliefs, ‘experiences’, ‘perceived insecurity’, ‘fear and anxiety’, ‘need for control’, ‘limited health literacy’, ‘over-informed’, ‘stage of life’, ‘maximization of length and/or quality of life’, and ‘acceptance of care recommended by the provider’. The dominance of the cancer-related articles in our data set seems to relate to including the terms overdiagnosis and overuse in our search strategy. These terms were often present in the title, abstract and keywords of the cancer-related articles. However, it is also possible that fear of cancer is associated with a higher use of low-value care, or that the topic of low-value care is more often discussed in the context of a life-threatening disease as cancer, compared to other diseases. Whether this means that demand for low-value care is more prevalent or more often studied in the field of cancer remains unclear and could be the subject of further research.

**Fig. 2:**
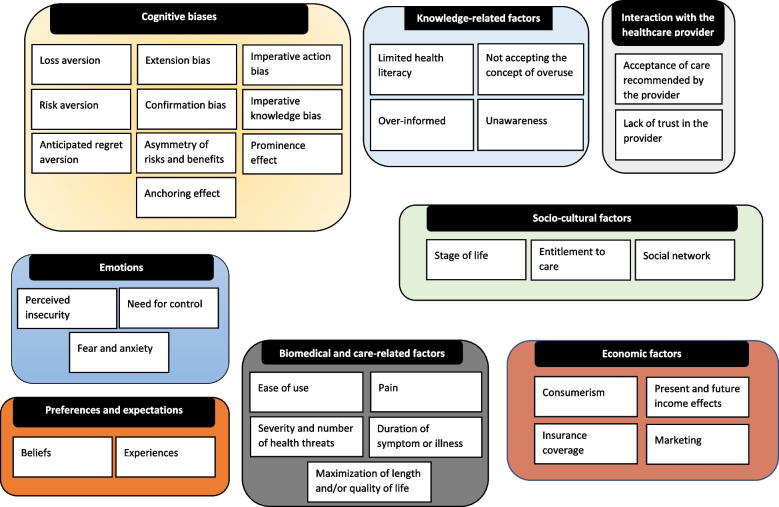
Overview of core and subthemes associated with patients’ demand for low-value care

The fourth limitation lies in the various themes being categorized in a categorical manner (see Fig. 2), which does not fully reflect the complexity of the processes in real life. Our manner of categorization suffices in theoretical situations like in the current review, where we aimed to provide insight in the variety of factors potentially associated with the demand for low-value care. But, in practice, it is challenging to make such rigid distinctions, because of the potential overlap and interactions between these demand-side factors. For example, the subthemes ‘perceived insecurity’, ‘fear and anxiety’, and ‘need for control’ are emotions that may coincide when dealing with uncertainty. Another example of overlap between themes concerns the core themes ‘cognitive biases’, ‘emotions’, and ‘expectations and preferences’. Cognitive biases, preferences and expectations can be formed by specific beliefs and experiences of patients but may also be inseparable from emotions that patients have. In fact, preferences and expectations are often emotionally laden, and emotions such as ‘fear and anxiety’, ‘perceived insecurity’, and ‘loss of control’ lie at the basis of patients’ beliefs to prefer unnecessary care. Furthermore, beliefs and cognitive biases could also have a mutual reinforcing relation. For instance, beliefs could be shaped by patients’ confirmation bias or by the asymmetry of risks and benefits bias, which in turn could reinforce a certain belief. For this reason, our construction of themes is not unambiguous and other researchers could have arrived at different categorizations.

Building forth on the previous point, categorization of the distinct themes can also be conducted in a different manner. Based on the description of the themes and their contexts it appears that some themes can encourage patients to demand low-value care in an intrinsic or extrinsic manner, or somewhere in between. For example, subthemes in the ‘cognitive biases’, ‘emotions’, ‘preferences and expectations’, ‘biomedical and care-related factors’ and ‘knowledge-related factors’ are more probable to drive patients’ demand from an intrinsic perspective [70, 71]. These themes can be characterized as demand drivers that primarily take place within the patient’s mind, and so result in the demand for low-value care. On the other hand, subthemes such as ‘social network’, and ‘marketing’ could fuel patients’ demand from an extrinsic perspective. In these subthemes the demand for low-value care originates from circumstances that revolve around the patient and incentivize patients to demand low-value care [26, 42, 74]. However, some subthemes are more difficult to categorize and can be perceived as more intrinsic or extrinsic forms of demand drivers depending on the context, such as ‘acceptance of care recommended by the provider’, ‘lack of trust in the provider’, entitlement to care’, and ‘consumerism’.

The sixth limitation concerns the synonyms used to define low-value care in our search strategy. To identify relevant literature, we used a broad set of terms that were commonly used for low-value care, such as: ‘unnecessary care’, ‘overuse’, ‘overdiagnosis’, ‘overtreatment’, and ‘cost ineffective care’. However, we did not include the term ‘inappropriate care’ in our study. While this term is used in some contexts, it may contribute to yield additional articles if applied in our search strategy. Despite this, we do expect the terms used in our search strategy to cover a majority of the articles related to low-value care in the literature. Therefore, we anticipate that inclusion of the term ‘inappropriate care’ will provide a small number of additional articles that do not provide additional themes or alter our findings significantly.

This study also provides recommendations for future research. In our study as well as in a large proportion of the included literature, the identified factors related to patients’ demand for low-value care were presented as separate, independent factors, but it is doubtful whether this is always the case [95]. Demand factors have been connected to other demand factors, but also to system and supply-related factors that contribute to low-value care usage [28, 37, 86]. For this reason, it has been argued that low-value care is a multifactorial interrelated complex problem [12, 28, 96] and that limiting the view on the problem of low-value care to single, independent determinants is not accurate. In this perspective, the identified factors can be seen as components of a more complex system in which patient and other factors influence each other in numerous ways, before resulting in the demand for low-value care by patients. Therefore, viewing patients’ demand from a complex systems perspective could provide more insight into importance of each factors, the interrelationships between factors, and how this affects patients’ demand in a specific context [97]. This type of research can offer valuable insights for the design of interventions to effectively reduce low-value care. Ideally, these interventions target not only the individual but also broader factors affecting patients’ demand for low-value care, including, for example, the healthcare system, healthcare providers and cultural norms and values of societal groups [12]. Such interventions are highly needed to combat the problem of low-value care and keep healthcare systems affordable in the future.

## Conclusions

This study identified a large variety of factors associated with patients’ demand for low-value care from the existing literature and, by categorizing and describing these factors, we provided initial understanding of how these factors may contribute to patients’ demand for low-value care. How important these demand-side factors are in specific contexts (e.g., disease, setting, country) and in relation to each other as well as system and supply-side factors requires further study. Nonetheless, the findings of this study may still be relevant for decision makers aiming to reduce patient demand for low-value care. For instance, physicians can use the findings from this overview to better understand the reasons for patients demanding low-value care and use these insights in their interactions with patients to steer them away from low-value care interventions. For policymakers, the findings of this study may be relevant when developing interventions to reduce low-value care use. For example, by developing policies that target or make patients aware of the identified economic, emotional or socio-cultural factors associated with low-value care demand. In this manner, policymakers may be able to help patients critically evaluate the origin and subsequently the appropriateness of their care needs. This may help patients refrain from demanding low-value care and encourage them to make high value-based care decisions. At the same time, physicians and policymakers need to be aware that more evidence is needed about the importance of and interactions between these factors in different contexts, and consider that efforts to reduce low-value care demand (in some) should not lead to the underuse of high-value care.

## Supplementary Information

Supplementary Material 1.

Supplementary Material 2.

Supplementary Material 3.

## Data Availability

Data is provided within the manuscript or supplementary information files (Appendix 1 and 2).
