# Supplementary material for: Factors associated with patients’ demand for low-value care: a scoping review
Source: BMC Health Serv Res. 2024 Dec 28;24:1656. doi: 10.1186/s12913-024-12093-7 (PMC11681654; doi:10.1186/s12913-024-12093-7)
Supplement: Supplementary file 1 — Supplementary Material 1. [file 12913_2024_12093_MOESM1_ESM.docx]

**Appendix 1 - Search Strategy**

**Table 1**

*Query strategy for the literature, conducted on the 25th of February 2022 in Embase*

| No. | Query | Results |
| --- | --- | --- |
| 1 | 'low-value care'/exp | 26 |
| 2 | (('low value' OR 'low-value') NEAR/3 ('care' OR 'health' OR 'servic*')):ti | 272 |
| 3 | (('low value' OR 'low-value') NEAR/2 ('care' OR 'health' OR 'servic*')):ab | 554 |
| 4 | 'overdiagnosis'/exp/mj OR 'overdiagnos*':ti | 1545 |
| 5 | ((('overuse' OR 'underuse') NEAR/3 ('health*' OR 'care' OR 'medicin*')):ti,ab) OR 'cost ineffective care':ti,ab OR 'unnecessary care':ti,ab | 1030 |
| 6 | #1 OR #2 OR #3 OR #4 OR #5 | 3207 |
| 7 | 'patient education'/exp OR (('patient*' NEAR/3 'educat*'):ti,ab) | 158329 |
| 8 | 'patient preference'/exp OR (('patient*' NEAR/3 'prefer*'):ti,ab) | 56457 |
| 9 | 'cognitive bias'/exp OR 'cognitive bias':ti | 16475 |
| 10 | ('clinical decision making'/exp/mj OR 'shared decision making'/exp) AND 'patient*':ti,ab | 11678 |
| 11 | 'regret*':ti OR 'uncertain*':ti OR 'health belief*':ti OR 'health norm*':ti OR 'trust*':ti OR 'complacen*':ti OR 'fear*':ti OR 'indifferen*':ti | 64330 |
| 12 | #7 OR #8 OR #9 OR #10 OR #11 | 301018 |
| 13 | #6 AND #12 | 201 |

* This symbol replaces one or more letters in the search.
 i.e. immun* captures hits with *immune, immunity, etc.*
NEAR/n NEAR/n searches for terms within the specified number of words from each other, in either direction. (therapy NEAR/5 sleep) looks for the word therapy within 5 words of sleep

/exp Explode will search with all subject headings below the main heading included and bring up all results listing any of these terms subject heading subheadings combinations. Command to search:/exp

/mj As major focus will only search for your chosen Emtree term as a main term. Main terms are chosen by Emtree indexers to be the primary focus of an individual article. Command to search: /mj

x:ti,ab The letter ‘’X’’ is the specific word searched for in the title or abstract. To search Title/abstract only use “:ab,ti.” For example: 'vaccine':ab,ti . This tag can also involve multiple search terms. For example: (opioid* OR narcotic*):ti,ab

**Table 2**

*Query strategy for the literature search, conducted from the 25th of February 2022 up to and including articles published on the 27^th^ of May in Embase*

| No. | Query | Results |
| --- | --- | --- |
| 1 | 'low-value care'/exp | 317 |
| 2 | (('low value' OR 'low-value') NEAR/3 ('care' OR 'health' OR 'servic*')):ti | 427 |
| 3 | (('low value' OR 'low-value') NEAR/2 ('care' OR 'health' OR 'servic*')):ab | 843 |
| 4 | 'overdiagnosis'/exp/mj OR 'overdiagnos*':ti | 1887 |
| 5 | ((('overuse' OR 'underuse') NEAR/3 ('health*' OR 'care' OR 'medicin*')):ti,ab) OR 'cost ineffective care':ti,ab OR 'unnecessary care':ti,ab | 1256 |
| 6 | #1 OR #2 OR #3 OR #4 OR #5 | 4165 |
| 7 | 'patient education'/exp OR (('patient*' NEAR/3 'educat*'):ti,ab) | 175282 |
| 8 | 'patient preference'/exp OR (('patient*' NEAR/3 'prefer*'):ti,ab) | 67730 |
| 9 | 'cognitive bias'/exp OR 'cognitive bias':ti | 22437 |
| 10 | ('clinical decision making'/exp/mj OR 'shared decision making'/exp) AND 'patient*':ti,ab | 17463 |
| 11 | 'regret*':ti OR 'uncertain*':ti OR 'health belief*':ti OR 'health norm*':ti OR 'trust*':ti OR 'complacen*':ti OR 'fear*':ti OR 'indifferen*':ti | 76279 |
| 12 | #7 OR #8 OR #9 OR #10 OR #11 | 350979 |
| 13 | #6 AND #12 | 283 |
| 14 | #6 AND #12 AND [25-02-2022]/sd NOT [01-07-2024]/sd | 95 |

**Table 3**

Query strategy for literature search, conducted on the 27^th^ of May in Scopus

| Search strategy in Scopus |
| --- |
| (INDEXTERMS("low-value-care" or "overdiagnosis") OR TITLE("low-value" W/3 ("care" or "health" or "servic*")) OR TITLE-ABS("low-value" W/2 ("care" or "health" or "servic*")) OR TITLE("overdiagnos*") OR TITLE-ABS(("overuse" or "underuse") W/3 ("health*" or "care" or "medicin*")) OR TITLE-ABS("cost ineffective care" or "unnecessary care")) |
| AND |
| (INDEXTERMS("patient education" or "patient preference" or "cognitive bias" ) OR TITLE-ABS("patient*" W/3 ("educat*" or "prefer*")) OR TITLE("cognitive bias" or "regret*" or "uncertain*" or "health belief*" or "health norm*" or "trust*" or "complacen*" or "fear*" or "indifferen*") OR (TITLE-ABS("patient*") AND INDEXTERMS("clinical decision making" or "shared decision making"))) |
| Results: 340 articles |
