# Supplementary material for: Factors associated with patients’ demand for low-value care: a scoping review
Source: BMC Health Serv Res. 2024 Dec 28;24:1656. doi: 10.1186/s12913-024-12093-7 (PMC11681654; doi:10.1186/s12913-024-12093-7)
Supplement: Supplementary file 3 — Supplementary Material 3. [file 12913_2024_12093_MOESM3_ESM.docx]

**Appendix 3 - *Core themes / Subthemes per article*^a^**

| **Study conducted by** | **Cognitive biases** | Asymmetry of risks and benefits | Extension bias | Imperative knowledge | Imperative action | Risk aversion | Anticipated regret aversion | Confirmation bias | Loss aversion | Anchoring effect | Prominence effect | **Preferences and expectations** | Beliefs | Experiences | **Emotions** | Perceived insecurity | Fear and anxiety | Need for control | **Knowledge-related factors** | Limited health literacy | Not accepting the concept of overuse | Unawareness | Over-informed | **Socio-cultural factors** | Stage of life | Social network | Entitlement to care | **Economic factors** | Consumerism | Marketing | Present and future income effects | Insurance coverage | **Biomedical and care-related factors** | Severity and number of health threats | Maximization of length and quality of life | Duration of symptom or illness | Pain | Ease of use | **Interaction with the healthcare provider** | Lack of trust in the provider | Acceptance of care recommended  by the provider |
| --- | --- | --- | --- | --- | --- | --- | --- | --- | --- | --- | --- | --- | --- | --- | --- | --- | --- | --- | --- | --- | --- | --- | --- | --- | --- | --- | --- | --- | --- | --- | --- | --- | --- | --- | --- | --- | --- | --- | --- | --- | --- |
| [[62](#_ENREF_62)] (Alber et al., 2017) | x |  | x |  |  | x |  |  |  |  |  | x | x |  | x | x |  |  | x |  |  | x |  |  |  |  |  | x | x | x |  |  |  |  |  |  |  |  | x | x |  |
| [[63](#_ENREF_63)] (Bishop et al., 2017) | x |  | x |  |  |  |  |  |  |  |  |  |  |  |  |  |  |  |  |  |  |  |  |  |  |  |  |  |  |  |  |  |  |  |  |  |  |  |  |  |  |
| [[22](#_ENREF_22)] (Born et al., 2019) | x |  | x |  |  |  |  | x | x |  |  |  |  |  |  |  |  |  | x |  | x |  |  |  |  |  |  |  |  |  |  |  |  |  |  |  |  |  |  |  |  |
| [[64](#_ENREF_64)] (Buist et al., 2016) | x | x |  |  |  |  |  |  |  |  |  | x | x |  |  |  |  |  |  |  |  |  |  |  |  |  |  |  |  |  |  |  |  |  |  |  |  |  |  |  |  |
| [[65](#_ENREF_65)] (Carpenter et al., 2015) |  |  |  |  |  |  |  |  |  |  |  | x | x |  |  |  |  |  |  |  |  |  |  |  |  |  |  |  |  |  |  |  |  |  |  |  |  |  |  |  |  |
| [[50](#_ENREF_50)] (Duffin et al., 2020) |  |  |  |  |  |  |  |  |  |  |  |  |  |  |  |  |  |  |  |  |  |  |  |  |  |  |  |  |  |  |  |  | x |  |  |  | x |  |  |  |  |
| [[66](#_ENREF_66)] (Edmeads et al., 1997) |  |  |  |  |  |  |  |  |  |  |  |  |  |  |  |  |  |  | x |  |  | x |  |  |  |  |  |  |  |  |  |  |  |  |  |  |  |  | x | x |  |
| [[42](#_ENREF_42)] (Ellen et al., 2021) | x |  |  |  |  |  | x | x |  | x |  |  |  |  | x | x | x | x | x | x |  |  | x | x |  | x |  | x | x |  |  |  | x |  | x |  |  |  |  |  |  |
| [[67](#_ENREF_67)] (Fifer et al., 2022) |  |  |  |  |  |  |  |  |  |  |  | x | x | x | x |  | x |  | x | x | x |  |  |  |  |  |  |  |  |  |  |  | x | x |  |  |  | x |  |  |  |
| [[68](#_ENREF_68)] (Gangathimmaiah et al., 2023) | x |  |  | x |  |  |  |  |  |  |  | x | x |  | x | x |  |  | x |  | x |  |  | x |  | x |  | x |  |  | x |  |  |  |  |  |  |  | x | x |  |
| [[69](#_ENREF_69)] (Gaver, 2022) | x | x |  |  |  |  |  |  |  |  |  | x | x | x | x |  | x |  |  |  |  |  |  |  |  |  |  |  |  |  |  |  |  |  |  |  |  |  | x |  | x |
| [[51](#_ENREF_51)] (Hersch et al., 2013) |  |  |  |  |  |  |  |  |  |  |  | x | x |  | x | x |  |  | x |  | x | x |  |  |  |  |  |  |  |  |  |  |  |  |  |  |  |  |  |  |  |
| [[70](#_ENREF_70)] (Hofmann, 2020) | x | x | x | x |  | x | x |  | x | x | x |  |  |  |  |  |  |  |  |  |  |  |  |  |  |  |  |  |  |  |  |  | x |  | x |  |  |  |  |  |  |
| [[71](#_ENREF_71)] (Hofmann, 2021) | x |  | x | x | x |  | x |  | x |  |  |  |  |  | x |  | x |  |  |  |  |  |  |  |  |  |  |  |  |  |  |  |  |  |  |  |  |  |  |  |  |
| [[72](#_ENREF_72)] (Hudgins and Rising, 2016) |  |  |  |  |  |  |  |  |  |  |  | x |  | x | x | x | x |  |  |  |  |  |  | x |  | x |  | x |  |  | x | x | x | x |  |  | x |  |  |  |  |
| [[53](#_ENREF_53)] (Jensen et al., 2020) | x |  | x |  | x |  |  |  |  |  |  | x | x |  | x | x | x |  |  |  |  |  |  |  |  |  |  |  |  |  |  |  |  |  |  |  |  |  |  |  |  |
| [[52](#_ENREF_52)] (Kangovi et al., 2013) | x |  |  | x |  |  |  |  |  |  |  |  |  |  |  |  |  |  |  |  |  |  |  |  |  |  |  |  |  |  |  |  |  |  |  |  |  |  | x | x |  |
| [[73](#_ENREF_73)] (Kazemi et al., 2024) | x |  | x |  |  | x |  |  |  |  |  | x | x | x |  |  | x |  |  |  |  |  |  | x | x |  |  |  |  |  |  |  | x | x | x |  |  |  |  |  |  |
| [[40](#_ENREF_40)] (Kherad et al., 2020) | x | x | x | x | x |  |  |  | x |  |  |  |  |  | x | x |  |  | x | x | x |  |  | x |  |  | x | x |  | x |  |  |  |  |  |  |  |  |  |  |  |
| [[5](#_ENREF_5)] (Kool et al., 2020) |  |  |  |  |  |  |  |  |  |  |  |  |  |  | x | x |  |  |  |  |  |  |  |  |  |  |  |  |  |  |  |  |  |  |  |  |  |  |  |  |  |
| [[74](#_ENREF_74)] (McCaffery et al., 2016) | x | x |  | x | x |  |  |  |  |  |  | x | x |  | x | x |  |  | x | x | x | x |  |  |  |  |  | x |  | x |  |  |  |  |  |  |  |  |  |  |  |
| [[46](#_ENREF_46)] (Mott et al., 2021) | x |  | x |  |  |  |  | x |  |  |  | x | x |  |  |  |  |  |  |  |  |  |  | x | x |  |  |  |  |  |  |  | x |  | x |  |  |  |  |  |  |
| [[75](#_ENREF_75)] (Munoz-Plaza et al., 2016) | x |  |  |  | x |  |  |  |  |  |  | x | x |  |  |  |  |  |  |  |  |  |  |  |  |  |  |  |  |  |  |  |  |  |  |  |  |  |  |  |  |
| [[26](#_ENREF_26)] (Pathirana et al., 2017) | x | x | x | x | x |  | x |  |  |  |  | x | x |  | x | x | x |  | x | x |  |  |  | x | x |  | x | x |  | x |  |  |  |  |  |  |  |  |  |  |  |
| [[76](#_ENREF_76)] (Paz-Martin and Arnal-Velasco, 2023) | x |  |  |  |  | x |  |  |  |  |  | x | x |  | x |  |  | x |  |  |  |  |  | x | x | x |  | x | x | x |  |  | x | x |  |  |  |  |  |  |  |
| [[49](#_ENREF_49)] (Perlman and Raeburn, 2021) |  |  |  |  |  |  |  |  |  |  |  |  |  |  |  |  |  |  |  |  |  |  |  | x | x |  |  |  |  |  |  |  | x |  |  | x |  |  |  |  |  |
| [[77](#_ENREF_77)] (Pickles et al., 2015) | x |  |  | x |  |  |  |  |  |  |  | x | x |  | x |  | x |  |  |  |  |  |  |  |  |  |  |  |  |  |  |  |  |  |  |  |  |  |  |  |  |
| [[78](#_ENREF_78)] (Pickles et al., 2021) | x |  | x |  |  |  |  | x |  |  |  |  |  |  |  |  |  |  | x | x |  |  |  |  |  |  |  |  |  |  |  |  |  |  |  |  |  |  |  |  |  |
| [[79](#_ENREF_79)] (Podder et al., 2019) | x | x |  |  |  |  |  |  |  |  |  | x | x |  | x | x | x |  | x | x |  |  |  |  |  |  |  |  |  |  |  |  |  |  |  |  |  |  |  |  |  |
| [[43](#_ENREF_43)] (Rapoport, 2008) |  |  |  |  |  |  |  |  |  |  |  |  |  |  |  |  |  |  |  |  |  |  |  |  |  |  |  | x |  |  | x |  | x | x |  |  |  |  |  |  |  |
| [[80](#_ENREF_80)] (Ropers et al., 2023) | x | x |  |  | x |  | x |  |  |  |  | x |  | x | x | x | x |  | x |  |  | x |  | x |  | x |  | x | x | x |  |  | x | x |  |  |  |  |  |  |  |
| [[39](#_ENREF_39)] (Rozbroj et al., 2021) | x | x |  | x |  |  |  |  |  |  |  | x | x | x | x | x |  |  | x | x | x | x | x |  |  |  |  |  |  |  |  |  | x |  | x |  |  |  |  |  |  |
| [[81](#_ENREF_81)] (Rudin et al., 2022) | x |  | x |  |  |  |  |  |  |  |  | x |  | x |  |  |  |  | x |  |  | x |  |  |  |  |  | x | x |  |  |  |  |  |  |  |  |  | x |  | x |
| [[82](#_ENREF_82)] (Salm et al., 2023) | x |  |  |  |  |  |  |  |  |  |  |  |  |  |  |  |  |  |  |  |  |  |  | x |  | x |  | x | x |  |  |  | x | x |  | x |  |  |  |  |  |
| [[47](#_ENREF_47)] (Scherer et al., 2020) |  |  |  |  |  |  |  |  |  |  |  | x | x |  |  |  |  |  |  |  |  |  |  | x | x |  | x |  |  |  |  |  | x | x |  |  |  |  |  |  |  |
| [[83](#_ENREF_83)] (Sicsic et al., 2018) | x | x | x |  |  |  |  |  |  |  |  | x |  | x |  |  | x |  |  |  |  |  |  |  |  |  |  |  |  |  |  |  | x | x | x |  |  |  |  |  |  |
| [[84](#_ENREF_84)] (Siedlikowski et al., 2018) |  |  |  |  |  |  |  |  |  |  |  | x | x | x |  |  | x |  |  |  |  |  |  |  |  |  |  |  |  |  |  |  |  |  |  |  |  |  |  |  |  |
| [[85](#_ENREF_85)] (Skolarus et al., 2021) | x |  |  |  | x |  |  |  |  |  |  |  |  |  |  |  | x |  |  |  |  |  |  | x |  | x |  |  |  |  |  |  | x | x |  |  |  |  |  |  |  |
| [[86](#_ENREF_86)] (Strobel et al., 2023) |  |  |  |  |  |  |  |  |  |  |  | x | x |  | x | x |  |  |  |  |  |  |  | x |  | x |  | x | x |  |  |  | x | x |  |  |  |  |  |  |  |
| [[87](#_ENREF_87)] (Sutkowi-Hemstreet et al., 2015) | x | x |  | x |  | x | x |  |  |  |  | x | x | x | x | x | x | x | x |  |  |  | x | x | x | x |  | x |  |  | x | x | x |  | x |  |  | x | x | x | x |
| [[48](#_ENREF_48)] (Taylor et al., 2018) | x |  |  |  |  | x |  |  |  |  |  | x | x |  | x |  | x |  | x | x |  |  |  | x | x |  |  |  |  |  |  |  |  |  |  |  |  |  |  |  |  |
| [[88](#_ENREF_88)] (van Egmond et al., 2019) |  |  |  |  |  |  |  |  |  |  |  | x |  | x | x | x | x |  |  |  |  |  |  |  |  |  |  |  |  |  |  |  |  |  |  |  |  |  | x | x | x |
| [[89](#_ENREF_89)] (van Egmond et al., 2021) |  |  |  |  |  |  |  |  |  |  |  |  |  |  |  |  |  |  |  |  |  |  |  |  |  |  |  |  |  |  |  |  |  |  |  |  |  |  | x |  | x |
| [[90](#_ENREF_90)] (Vercellini et al., 2015) | x | x |  |  |  |  |  |  |  |  |  |  |  |  |  |  |  |  |  |  |  |  |  |  |  |  |  |  |  |  |  |  |  |  |  |  |  |  |  |  |  |
| [[44](#_ENREF_44)] (Wammes et al., 2014) |  |  |  |  |  |  |  |  |  |  |  |  |  |  | x | x |  |  |  |  |  |  |  | x |  |  | x | x | x |  |  |  |  |  |  |  |  |  | x | x |  |
| [[91](#_ENREF_91)] (Wang et al., 2021) | x | x |  | x |  | x |  |  |  |  |  |  |  |  | x | x |  |  | x |  | x |  |  | x | x |  |  |  |  |  |  |  |  |  |  |  |  |  | x | x |  |
| [[45](#_ENREF_45)] (Yin et al., 2019) |  |  |  |  |  |  |  |  |  |  |  |  |  |  |  |  |  |  |  |  |  |  |  |  |  |  |  | x |  |  | x |  |  |  |  |  |  |  |  |  |  |
| Coverage of themes in articles | 31 | 13 | 13 | 11 | 8 | 7 | 6 | 4 | 4 | 2 | 1 | 27 | 22 | 11 | 24 | 18 | 17 | 3 | 18 | 9 | 8 | 7 | 3 | 18 | 9 | 9 | 4 | 16 | 8 | 6 | 5 | 2 | 18 | 11 | 7 | 2 | 2 | 2 | 11 | 8 | 5 |

Note: ^a^ Themes highlighted in bold are core themes. Themes that were not highlighted are subtheme

**Appendix 3 - *Core themes / Subthemes per article*^a^ (Continued)**

**Continued)**
